# Barriers and facilitators to evidence-use in program management: a systematic review of the literature

**DOI:** 10.1186/1472-6963-14-171

**Published:** 2014-04-14

**Authors:** Serena Humphries, Tania Stafinski, Zubia Mumtaz, Devidas Menon

**Affiliations:** 1 School of Public Health, University of Alberta, Room 3021 Research Transition Facility 8308 114 Street, Edmonton, Alberta T6G 2V2, Canada; 2 Health Technology and Policy Unit, School of Public Health, University of Alberta, Room 3028 Research Transition Facility, 8308 114 Street, Edmonton, Alberta T6G 2V2, Canada; 3 School of Public Health, University of Alberta, 3–309 Edmonton Clinic Health Academy, 11405 87 Avenue, Edmonton, Alberta T6G 1C9, Canada; 4 School of Public Health, University of Alberta, Room 3032 Research Transition Facility, 8308 114 Street, Edmonton, Alberta T6G 2V2, Canada

**Keywords:** Decision-making, Evidence-use, Barriers, Facilitators, Program management

## Abstract

**Background:**

The use of evidence in decision-making at the program management level is a priority in health care organizations. The objective of this study was to identify potential barriers and facilitators experienced by managers to the use of evidence in program management within health care organizations.

**Methods:**

The authors conducted a comprehensive search for published, peer-reviewed and grey literature that explores the use of evidence in program management. Two reviewers selected relevant studies from which data was extracted using a standard data abstraction form and tabulated for qualitative analysis. The results were summarized through narrative review. The quality of the included studies was assessed using published criteria for the critical appraisal of qualitative, quantitative and mixed methods research.

**Results:**

Fourteen papers were included in the review. Barriers and facilitators were categorized into five main thematic areas: (1) Information, (2) Organization – Structure and Process, (3) Organization – Culture, (4) Individual, and (5) Interaction.

**Conclusion:**

This paper reviews the literature on barriers and facilitators to evidence-informed decision-making experienced by program management decision-makers within health care organizations. The multidimensional solutions required to promote evidence-informed program management can be developed through an understanding of the existing barriers and facilitators of evidence-use.

## Background

Health care organizations are complex and comprised of highly trained individuals whose responsibilities include management of conflicting demands for services. Consequently, the types of decisions that must be made are often complicated and they are different, depending on the level within the organization. These could range from governance-related decisions by the Board of Directors or Trustees to decisions on treatment options for individual patients by a physician.

Over the last two decades (and particularly in Canada), clinicians have begun to take on increased roles in management structures, mainly because they are often viewed as major “drivers” of health care expenditures. This has resulted in greater involvement in budget and operational decisions, as well as the development of the concept of program management. It is defined as “a type of management structure in which services are grouped into programs by medical specialty, specific diagnosis or populations groups” [1], pp. 186–188. Program managers are responsible for the design and implementation of specific health services programs to achieve their objectives. This includes planning, dealing with complex interdependencies, service integration and appropriate pacing of the program. For example: include establishing a program that provides dialysis in hospital or at home or introducing community-based mental health services in a regional health authority. In recent years, the demand that program management –related decisions reflect the best available evidence has heightened. This may be explained by the promotion of evidence-based decision-making in healthcare [2].

Many organizations have established evidence-informed processes for making acquisition decisions around new equipment, supplies, and pharmaceuticals. However, at the program management level, such processes are less well-developed [3,4]. In fact, to date, published studies have demonstrated a lack of evidence-informed decision-making, as well as limited research aimed at formulating best practice approaches [5-7].

This may be, in part, attributed to perceived barriers to the use of evidence by program level decision-makers. Clinicians involved in determining the most appropriate patient care options face different barriers, such as patient conditions that may fall outside of clinical practice guidelines or rare conditions for which guidelines have not yet been developed. In contrast, those faced by program managers often relate to complex organizational issues involving multiple stakeholder communities with competing interests. Nonetheless, the need to find ways of overcoming these barriers has been underscored by a widely held view that evidence-informed program management decision-making may serve to improve the acceptability of decisions to such stakeholder communities [5].

Evidence-informed program management decision-making requires two sets of skills: 1) those for identifying and critically assessing the evidence and 2) those for applying it to their local context in a way that reflects an awareness and understanding of factors potentially affecting uptake, implementation or sustainability of the evidence within a complex setting [2]. In doing so, the users of that evidence must recognize the varying degrees of rigour and quality of evidence applied. Whether such skill sets exist within organizations and reasons for their presence or absence have yet to be fully explored.

### Objective

The purpose of this review of existing empirical studies was to identify potential barriers and facilitators to evidence-informed decision-making experienced by program management decision-makers within health care organizations.

### Methods

A comprehensive review of published empirical studies of program management was performed following best practice guidelines for conducting systematic reviews in health services research [8].

#### *Search strategy*

The following bibliographic databases were searched for relevant English language peer-reviewed and grey literature published between October 2000 and December 2011: PubMed (MEDLINE and non-MEDLINE references), the Cochrane Library, the Centre for Reviews and Dissemination (DARE, NHS EED and HTA), EMBASE, ProQuest Dissertations & Theses, CINAHL, Web of Science, and ABI Inform. There is no standard definition of program management in the literature. Consequently, the types of initiatives of interest to this review may have been indexed using a variety of terms. Therefore, the search strategy applied to the databases included a broad range of controlled vocabulary terms, such as the Medical Subject Headings (MeSH) terms: ‘decision-making’ and ‘program development’, as well as additional keywords such as ‘evidence-informed’, ‘knowledge utilization’, ‘barriers’ and ‘facilitators’. MEDLINE was also searched for papers by key authors in the field. Grey literature was identified through the following sources: NYAM Grey literature collection, The Campbell Collaboration Library of Systematic Reviews, Quebec Population Health Research Network’s KU-UC database, and McMaster University Health Information Research Unit’s KT + database. For comprehensiveness, references in relevant papers were scanned to identify additional citations. Full details of the search terms and sources used are included in Additional file 1.

#### *Study eligibility criteria*

Study selection was completed by two reviewers, who independently scanned the titles and abstracts of citations identified through the search for inclusion in the review. Empirical studies in the English language exploring the use of evidence in program delivery, such as design, management or implementation, were included. Studies limited to clinical or health policy decision-making at levels other than that of a program were excluded. In order to compare countries with similar economies and socio-demographics to Canada, only studies examining evidence-use in OECD (Organization for Economic Co-operation and Development) countries were included. Finally, studies discussing non-medical services were excluded. See Table 1 for a summary of the inclusion and exclusion criteria.

**Table 1:** Study inclusion and exclusion criteria

**Inclusion criteria**	**Exclusion criteria**
• Date of Publication (October 2000-December 2011)	• Date of Publication (Prior to October 2000)
• English Language	• Non-English Language
• Empirical Study	• Non-Empirical Study
• Evidence Use in Program Management	• Evidence Use in Clinical Decision-Making
• OECD Country	• Evidence Use in Health Policy Decision-Making
	• Not OECD Country
	• Non-Medical Services

#### *Data collection and analysis*

For each selected study, information on study design, decision-making context, location, sector, type of decision-maker, and findings was extracted using a standard data abstraction form. For the purposes of this review, the Canadian Health Services Research Foundation’s decision-maker classification was used: Policy Makers defined as politicians and advisors, civil servants, board members, special interest groups and the public; Managers defined as institutional or regional Chief Executive Officers, program managers, clinical managers and management consultants; Service professionals defined as physicians, nurses, social workers, councilors and their associations [9]. One reviewer extracted data from all of the studies. However, for a random sample (10%), data were extracted by a second reviewer to assess reliability.

The data collected were entered into tables to facilitate qualitative analyses. Specifically, thematic analysis was used. This involved systematic identification of recurring themes. An initial list of codes for barriers and facilitators of evidence-use was prepared a priori by the research team based on expert opinion and a preliminary review of the relevant literature and then applied to a sample of eight of the included studies and revised as needed. The codes for the barriers and facilitators were reviewed by the study team to identify any gaps and were then categorized by theme. The findings from all of the included studies were coded based on the identified themes and analyzed quantitatively. The results were then summarized through narrative review [10].

The quality of studies was assessed using published criteria for critically appraising qualitative, quantitative and mixed methods research through a single tool [11]. Such criteria examine the methodological quality of the studies in order to judge their trustworthiness, value and relevance. For qualitative studies, the criteria examined data sources, data analysis, research context and researcher influence, while the quantitative criteria first categorized the studies as randomized, non-randomized or descriptive and then applied appropriate methodological criteria such as sampling strategy, measurement and response rate. Mixed method studies were assessed based on relevance of mixed method design, integration of methods and limitations. For each of the included studies, the relevant quality questions were asked and the studies were scored as “Yes” if they clearly met the criteria, “No” if they clearly did not meet the criteria, “Unclear” if it could not be determined by the reporting whether they met the criteria, or “Not Applicable” if the specific quality question did not apply to the study design.

## Results

**Figure 1:**
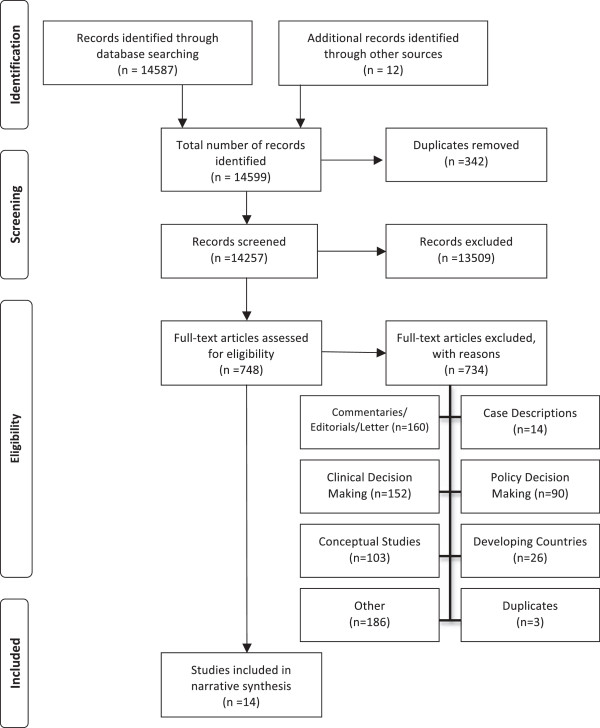
PRISMA flow diagram – search summary.

The literature search identified a total of 14,587 studies. Once duplicates were removed, 14,257 remained. The titles and abstracts of these references were reviewed and 748 were selected for full text review. As described in the search strategy, due to a lack of standard definition of program management in the literature, the broad search strategy employed for this review resulted in a large number of potential publications, the majority of which (13,509) were eliminated in the initial review of titles and abstracts, as they were not on the topic of evidence use in program management decision-making. Of the remaining 748 studies, 734 were excluded for the following reasons: commentaries, editorials, or letters (160); studies of clinical decision-making (152); conceptual studies (103); case descriptions (14); studies of policy decision-making (90); studies in developing countries (26); duplicates (3); and other (186). Ultimately, 14 papers involving 3,584 decision-makers met the inclusion criteria. Since themes were consistent across papers, it was concluded that saturation had been achieved and there was no need for further searching [12,13]. Figure 1 is a PRISMA flow diagram illustrating the search results (adapted from [14]).

### Overall characteristics of included studies

#### *Study design*

Table 2 summarizes the methods and designs of the included studies. Five of the included studies were qualitative in their design, five studies were quantitative, and four studies were mixed methods. The methods used in the studies included interviews (9), focus groups (3), documentation review (2), telephone surveys (3), written surveys (4) and case studies (1). Six employed multiple methods to address the research question, making the total number of methods used in the studies greater than the number of included studies.

**Table 2:** Included studies – characteristics

**Primary author**	**Publication year**	**Study methods**	**Study design**	**Country**	**Setting**	**Participants**
1 Belkhodja	2007	Telephone Survey	Quantitative	Canada	Ministries, Health Authorities, Hospitals	928 decision-makers (managers and professionals)
2 Bowen	2009	Interviews and Focus Groups	Quantitative	Canada	Health Authorities	205 decision-makers (senior managers, middle managers and board members)
3 Dobbins	2007(a)	Interviews	Quantitative	Canada	Public Health Units	16 decision-makers (6 program mangers, 6 directors, 1 Medical Officer of Health)
4 Dobbins	2001	Telephone Survey and Questionnaire	Quantitative	Canada	Public Health Units	141 decision-makers (medical and associate medical officers of health, program directors, program managers)
5 Dobbins	2007(b)	Telephone Survey	Quantitative	Canada	Community-based health organizations	92 decision-makers (from any level from CEO to front-line clinicians, senior planners)
6 Farmer	2001	Interviews	Quantitative	Scotland	Health Authorities	15 decision-makers (7 Directors’and 8 physician advisors)
7 Ham	2003	Interviews, Questionnaires, Case Studies	Qualitative and Quantitative	United Kingdom	Health Authorities	257 decision-makers (152 managers, 44 medical specialists, 21 nurses, 12 administrative and clerical staff, 12 GPs, 16 other) 4 case studies
8 Higgins	2011	Interviews	Qualitative	Canada	Health Authorities	21 decision-makers (16 front-line staff 5 managers)
9 Jbilou	2007	Survey	Qualitative	Canada	Health Organizations (Hospitals, Health Authorities, Ministries, Agencies)	942 decision-makers (managers, professionals, in ministries, hospitals, boards and councils)
10 McDiarmid	2007	Telephone Interview	Qualitative and Quantitative	Canada	Hospitals	21 decision-makers (16 front-line staff 5 managers)
11 Mitton	2004	Interviews and Focus Groups	Qualitative	Canada	Health Authority	25 decision-makers (senior managers, clinicians)
12 Niedzwiedzka	2003	Survey, Interviews, Focus Groups, Document Review	Qualitative and Quantitative	Poland	Hospitals and Departments of Health	815 decision-makers (hospital CEOs, medical directors, head nurses, directors) (#s for interviews and focus groups unknown)
13 Weatherly	2002	Survey, Interviews, Document Review	Qualitative and Quantitative	United Kingdom	Health Authorities	102 Health Authorities (78 decision makers N 68 coordinators, 10 leaders)
14 Wilson	2001	Online Survey	Quantitative	Canada	Community-based health organizations	25 decision-makers (Executive Directors)

#### *Location and decision-making setting*

The majority of the included studies were conducted in Canada (10). Studies were also from the United Kingdom (3), and Poland (1). The decision-making settings were health authorities (8), public health units (2), hospitals (4), community-based health organizations (2), and other health care organizations or jurisdictions (3).

### Type of decision-makers

The studies comprised a wide range of decision-makers such as senior managers, directors, Chief Operating Officers, clinicians and other front-line staff. Table 2 summarizes the main characteristics of the studies.

### Quality of included studies

Adhering to published guidelines for systematic reviews, the quality of the included studies was assessed using a mixed-methods assessment tool. A single critical appraisal tool enabled the quality of the quantitative, qualitative and mixed-methods studies to be assessed, compared and summarized using a single tool. Table 3 summarizes the quality assessment of the included studies. Studies are organized by design (Qualitative, Quantitative, Mixed Method) and the relevant quality questions are reported for each study. Based on responses to questions comprising the critical appraisal criteria for mixed methods study designs [11], the overall quality of the studies was fair. As summarized in Table 3, the methods in all of the included studies were poorly described, leading to an “unclear” rating for at least one of the methodological criteria assessment questions. Among the qualitative studies, few triangulated findings through the use of multiple methods for addressing the same question, few performed member checking to ensure accuracy in the responses collected from participants, and few mentioned sampling until saturation was reached. Among the quantitative studies, response rates were generally acceptable, but the representativeness of the sample populations was unclear, and validity of the measurement instruments was not adequately addressed in all of the studies. Among the mixed method studies, half did not provide a rationale for a mixed method design or discuss how the qualitative and quantitative data were meaningfully brought together to explore the research questions.

**Table 3:** Included studies – quality assessment

		**Qualitative**					**Qualitative**					**Mixed method**			
**Type-of-study**	**Methodological-quality-criteria**	**Bowen **[22]	**Dobbins **[15]	**Farmer **[16]	**Higgins **[17]	**Mitton **[18]	**Belkhodja **[27]	**Dobbins **[24]	**Dobbins **[26]	**Jbilou **[28]	**Wilson **[21]	**Ham **[25]	**Niedzwiedzka **[19]	**Weatherly **[20]	**McDiarmid **[23]
Screening Questions	Are there clear qualitative and quantitative research questions (or objectives), or a clear mixed methods question (or objective)?	Yes	Yes	Yes	Yes	Yes	Yes	Yes	Yes	Yes	Yes	Yes	Yes	Yes	Yes
	Do the collected data allow the research question (objective) to be appropriately addressed?	Yes	Yes	Yes	Unclear	Unclear	Unclear	Unclear	Yes	Unclear	Unclear	Yes	Yes	Unclear	Unclear
1. Qualitative	1.1. Are the sources of qualitative data (archives, documents, informants, observations) relevant to address the research question (objective)?	Yes	Yes	Yes	Unclear	Unclear	***Not Applicable***	***Not Applicable***	***Not Applicable***	***Not Applicable***	***Not Applicable***	Unclear	Yes	Unclear	Yes
	1.2. Is the process for analyzing qualitative data relevant to address the research question (objective)?	Yes	Yes	Yes	Unclear	Unclear	***Not Applicable***	***Not Applicable***	***Not Applicable***	***Not Applicable***	***Not Applicable***	Unclear	Unclear	Unclear	Unclear
	1.3. Is appropriate consideration given to how findings relate to the context, e.g., the setting, in which the data were collected?	Unclear	Unclear	Unclear	Unclear	Unclear	***Not Applicable***	***Not Applicable***	***Not Applicable***	***Not Applicable***	***Not Applicable***	Unclear	Unclear	Unclear	Unclear
	1.4. Is appropriate consideration given to how findings relate to researchers’ influence, e.g., through their interactions with participants?	No	No	No	No	No	***Not Applicable***	***Not Applicable***	***Not Applicable***	***Not Applicable***	***Not Applicable***	Unclear	Unclear	Unclear	Unclear
4. Quantitative Descriptive	4.1. Is the sampling strategy relevant to address the quantitative research question (quantitative aspect of the mixed methods question)?	***Not Applicable***	***Not Applicable***	***Not Applicable***	***Not Applicable***	***Not Applicable***	Yes	Yes	Yes	Yes	Yes	Unclear	Yes	Yes	Yes
	4.2. Is the sample representative of the population understudy?	***Not Applicable***	***Not Applicable***	***Not Applicable***	***Not Applicable***	***Not Applicable***	Unclear	Unclear	Unclear	Unclear	Unclear	Unclear	Unclear	Yes	Unclear
	4.3. Are measurements appropriate (clear origin, or validity known, or standard instrument)?	***Not Applicable***	***Not Applicable***	***Not Applicable***	***Not Applicable***	***Not Applicable***	Unclear	Yes	Yes	Unclear	Yes	Unclear	Yes	Unclear	Yes
	4.4. Is there an acceptable response rate (60% or above)?	***Not Applicable***	***Not Applicable***	***Not Applicable***	***Not Applicable***	***Not Applicable***	Yes	Yes	Yes	Yes	No	Unclear	Unclear	Yes	No
5. Mixed Methods	5.1. Is the mixed methods research design relevant to address the qualitative and quantitative research questions (or objectives), or the qualitative and quantitative aspects of the mixed methods question (or objective)?	***Not Applicable***	***Not Applicable***	***Not Applicable***	***Not Applicable***	***Not Applicable***	***Not Applicable***	***Not Applicable***	***Not Applicable***	***Not Applicable***	***Not Applicable***	Yes	Unclear	Yes	Unclear
	5.2. Is the integration of qualitative and quantitative data (or results) relevant to address the research question (objective)?	***Not Applicable***	***Not Applicable***	***Not Applicable***	***Not Applicable***	***Not Applicable***	***Not Applicable***	***Not Applicable***	***Not Applicable***	***Not Applicable***	***Not Applicable***	Yes	Unclear	Yes	Unclear
	5.3. Is appropriate consideration given to the limitations associated with this integration, e.g., the divergence of qualitative and quantitative data (or results) in a triangulation design?	***Not Applicable***	***Not Applicable***	***Not Applicable***	***Not Applicable***	***Not Applicable***	***Not Applicable***	***Not Applicable***	***Not Applicable***	***Not Applicable***	***Not Applicable***	Unclear	Unclear	Yes	Unclear

Barriers and facilitators identified in the literature were categorized into five themes: (1) Information, (2) Organization – Structure and Process, (3) Organization – Culture, (4) Individual, and (5) Interaction. Those relating to the production or use of information were classified as informational, such as dissemination strategies or perceived relevance of available research. Organizational barriers and facilitators including organizational systems, supports or procedures were classified as organizational structure and process. Those related to the values, principles or beliefs of the organization, such as visibility of evidence use within the organization, were classified as organizational culture. Individual barriers and facilitators associated with research knowledge or formal training were classified as individual skills. Those describing contact or relationships between researchers and decision-makers were classified as interaction. Each of the themes is discussed in the following sections, first for barriers and then for facilitators of evidence use.

### Barriers to evidence use

The majority (12) of studies identified barriers. In general, barriers experienced by managers were informational (10), including “availability of relevant research” [6,15], and organizational structure and process-related (10), including “problems linked to the complex nature of organizational decision-making and the challenges of integrating evidence therein” [16], p. 267. Seven studies reported individual barriers to evidence use and seven studies reported organizational culture as a barrier. Interaction between researchers and decision-makers was also mentioned in one of the studies. Table 4 provides a summary of the barriers identified in each theme for the included studies.

**Table 4:** Barriers to evidence use: summary of themes

**Primary author**	**Publication year**	**Information**	**Organization (structure & process)**	**Organization (culture)**	**Individual**	**Interaction**
1 Belkhodja	2007	X				
2 Bowen	2009	X	X	X	X	
3 Dobbins	2007(a)					
4 Dobbins	2001	X				
5 Dobbins	2007(b)	X	X		X	
6 Farmer	2001	X	X	X	X	
7 Ham	2003		X	X		
8 Higgins	2011	X	X	X		
9 Jbilou	2007					
10 McDiarmid	2007	X	X			
11 Mitton	2004	X	X	X	X	
12 Niedzwiedzka	2003	X	X		X	
13 Weatherly	2002	X	X	X	X	
14 Wilson	2003		X	X	X	X

(X indicates that the article was a source of evidence for the theme).

Within each theme, different specific types of barriers to evidence use were identified. Table 5 describes the types of barriers experienced by decision-makers for each of the barrier themes, which are subsequently explored in detail in the following section.

**Table 5:** Barriers to evidence use: summary of types by theme

**Barrier theme**	**Types of barrier**
Information	• Irrelevance of research
	• Unclear definition of evidence
	• Negative perceptions of research
	• Limited access to information
	• Mismatch of research to complex reality
	• Time required to produce research
	• Excess quantity of information
Organization (Structure and Process)	• Time limitations
	• Lack of internal research resources
	• Human resource constraints
	• Financial constraints
	• Lack of data and systems
	• Deficient planning processes
	• Absence of processes
	• Poor support from senior management
	• Rigid program silos
	• Competing priorities
	• Poor communication
Organization (Culture)	• Decision-making
	• Crisis management
	• Resistance to change
	• Politically influenced decisions
	• Challenging the promotion of evidence use
Individual Skills	• Research literacy
	• Research utilization
	• Management
Interaction	• Decision-maker/researcher gap
	• Mutual mistrust

#### *Barriers: information*

Program management decision-makers in health care organizations require a variety of information to inform decisions. This can include research findings, local evaluation results, expert opinion or professional experience [17-20]. The most frequently cited barrier to evidence-use that emerged from our analysis was information. The most frequently cited barriers to evidence-use among health care organization decision-makers relate to perceptions of the information generated through academic research. Decision-makers perceived a lack of relevant research, particularly research that could be used to make decisions at the local level [15,17-21]. Mitton and Patten report that a “barrier to the application of evidence in priority-setting was the difficulty in applying evidence in the local context” [18], p. 148. Overall, negative perceptions of research by decision-makers were also identified as a barrier to the use of evidence [16,17,19,22]. In Niedzwiedzka’s study of health care decision makers, for example, “Only 15% of respondents thought that research results had significant influence on practice in health care, and only 3.2% perceived developments in scientific knowledge as having an input in their area of decision making” [19], p. 108. Two studies also found that research that does not reflect the complex reality of the health care decision-making environment was a barrier to evidence-use [18,20]. Confusion regarding what constitutes evidence contributed to a lack of evidence-use by decision-makers [17-20]. Too much information [17,20] and difficulty accessing relevant information [15,19,20,23] were also identified as barriers. The amount of time it takes for research to be completed in order to inform a decision was also perceived as a barrier to evidence-use [23,24].

#### *Barriers: organization – structure and process*

An organization’s structure and processes emerged as an important barrier to the uptake of evidence in program management. The most frequently cited organizational barriers to evidence-use were time (6) and internal resource constraints (6). Evidence use in program management is challenged by a lack of time [15,18-22] and internal resources for research [19-23]. Bowen et al. report:

*“Lack of time and resources emerged as key barriers. Under-resourcing was described as resulting in poor decisions, …an inability to allocate resources to research or evidence-related positions and (perhaps most importantly) workload pressures that were described as actively working against the thoughtful reflection essential for [evidence-informed decision making]” *[22], p. 93.

Internal resource constraints included human resource constraints [20-22,25], financial constraints [15,19,23], workload issues such as competing priorities [22], and a lack of organizational data and systems [20,23,24]. Organizational leadership, especially a lack of senior management support for evidence-informed decision-making [17,22], a paucity of processes within organizations to incorporate evidence into program management decisions [16,18], and a lack of formal planning processes [16,22] were also identified as barriers to evidence use. Poor communication within an organization, between and across levels, as well as programs operating in isolation from other programs within the same organization further inhibited the use of evidence [22].

#### *Barriers: organization – culture*

Organizational culture was identified as a barrier in program management within health care organizations, particularly the decision-making culture of organizations [16-18,21] and crisis management culture of health care [16,18,22]. One study suggested that a “cultural shift [was] thought to be required to begin to use evidence” [18], p. 148. The highly politicized environment within which health care organizations undertake program management also contributed to challenges experienced by decision-makers in using evidence to inform decisions [20,22]. An overall resistance to change [22,25] and challenges in implementing change within health care organizations [22] were also barriers to evidence-use identified by decision-makers.

#### *Barriers: individual*

Decision-makers in health care organizations also experienced barriers to evidence use at the individual level. A deficit in the skills and experience of decision-makers in research literacy and research utilization, and a lack of formal management training were expressed as barriers to evidence-use in program management [15,16,18-22]. According to Wilson et al., referring to a survey of executive directors in community-based organizations:

*“Capacity was lowest for the domains related to: acquiring research (subsection I); assessing the reliability, quality, relevance, and applicability of research evidence (subsections III and IV); and summarizing results in a user-friendly way”*[21], p. 3*.*

#### *Barriers: interaction*

One of the included studies also identified issues related to the interaction between researchers and decision-makers as barriers to the use of evidence in health care organizations. The gap between researchers and decision-makers, in terms of a lack of contact and mutual understanding was identified as a barrier to evidence use [21].

### Facilitators of evidence use

The majority (10) of the included studies identified facilitators of evidence use for program management. The majority of facilitators of evidence-use experienced by managers were informational (10), for example,

*“Public health decision-makers value the use of systematic reviews to facilitate the decision-making process. They indicated that systematic reviews were particularly useful because they integrate the results of many studies into one, which allows them to bypass the stage of looking at individual studies. This saves them time and gives them more confidence knowing their decisions are based on the culmination of many studies instead of just a few”*[26], p. 159.

Organizational structure and process or organizational culture were identified as facilitators of evidence use in eight of the studies. One study concluded that evidence use “in health service organizations was more complex and much more sensitive to organizational factors and processes than previous studies seemed to affirm” [27], p. 407. Interaction between researchers and decision-makers was found to be a facilitator of evidence use in five of the studies. Four studies reported individual skills as facilitators of evidence use. Table 6 provides a summary of the facilitators identified by theme for the included studies.

**Table 6:** Facilitators of evidence use: summary of themes

**Primary author**	**Publication year**	**Information**	**Organization (structure & process)**	**Organization (culture)**	**Individual**	**Interaction**
1 Belkhodja	2007	X	X	X	X	X
2 Bowen	2009					
3 Dobbins	2007(a)	X				X
4 Dobbins	2001	X		X	X	
5 Dobbins	2007(b)	X				X
6 Farmer	2001	X	X	X		
7 Ham	2003			X	X	X	
8 Higgins	2011						
9 Jbilou	2007	X	X	X		X	
10 McDiarmid	2007						
11 Mitton	2004	X	X				
12 Niedzwiedzka	2003	X				X	
13 Weatherly	2002	X	X	X			
14 Wilson	2011	X	X	X	X		

(X indicates that the article was a source of evidence for the theme).

Within each theme, different specific types of facilitators of evidence use were detailed. Table 7 describes the types of facilitators identified by decision-makers for each of the facilitator themes.

**Table 7:** Facilitators of evidence use: summary of types by theme

**Facilitator theme**	**Types of facilitator**
Information	• Access to information
	• Complex intervention evaluation methods
	• Targeted dissemination
Organization (Structure and Process)	• Intra-organizational linkages
	• Expertise in research utilization
	• Processes for integration of evidence
	• Administrative support
	• Operational data availability
Organization (Culture)	• Supporting evidence use
	• Human resources training and rewards
	• Inter-organizational collaboration
	• Visible research utilization
Individual Skills	• Researcher and decision-maker focus on application
Interaction	• Contact between researchers and decision-makers
	• Mutual respect

#### *Facilitators: information*

The studies included in this review identified information related facilitators of evidence-use experienced by decision-makers in health care organizations. Access to information [15,16,19-21,24,28] as well as targeted dissemination of research findings to decision-makers [15,18,20,26,27] were identified as important facilitators of evidence use. Decision maker’s access to information was highlighted by one study which concluded that it was important for:

*“research-producing organizations knowing not only who their target audience(s) are and what their needs are concerning research evidence, but also what questions require answers, and what kind of answers are optimal for different types of decisions”*[15] p. 9*.*

The advancement of research methods to meet the needs for evaluating complex interventions [16] was also identified as a facilitator of evidence-informed decision-making.

#### *Facilitator: organizational – structure and process*

Organizational structure and processes also emerged as facilitators of evidence use for program management. Facilitators of evidence-use that relate to the structure and processes of health care organizations included administrative support [16,25,28] and intra-organizational linkages that promote knowledge sharing across the organization [16,18,21,27,28]. Developing internal expertise on research utilization [16,20,21,27] and formalizing the integration of evidence into decision-making processes [18,20,21], were also facilitators of evidence-use. The importance of organizational structure and process to evidence use is highlighted in one study which reports that “developing formal and informal linkage mechanisms, and creating policies that foster user’s experience in research are key factors to increase research utilization” [27], p. 406. An additional facilitator to evidence-use at the organizational level included the availability of operational data to support decision-making [16,20].

#### *Facilitator: organizational - culture*

The studies included in this review also reported that evidence-informed decision-making is influenced by an organization’s culture. An organizational culture that is supportive of evidence use, providing required supports and demonstrating through action that evidence-use is valued [16,21,24,27,28] and through providing necessary human resources, training and rewards for evidence-use [20,21,25] were seen as facilitators of evidence-use in health care organizations. As one study’s authors concluded, “making research one of the main pillars of the organizational culture of health service organizations” is a critical success factor to increasing evidence use in decision-making [27], p. 406. Ensuring the visibility of research utilization [27,28] within the organization was also identified as a facilitator. In addition, evidence-use within health care organizations was facilitated through inter-organizational collaboration and the sharing of information, expertise and experiences between organizations [27,28].

#### *Facilitator: individual*

Facilitators to evidence-informed decision-making were also identified at the individual level. Individual skill building for decision-makers in research literacy, research utilization and research application was identified as a facilitator of evidence use within health care organizations. The use of evidence for decision-making was also facilitated through the building of individual researcher’s skills to produce evidence that is useful to decision-makers and disseminate evidence to decision-makers more effectively [21,24,25,27]. For example, in one study, a decision-maker’s “experience in research strongly explained research result use among health managers” [27], p. 406.

#### *Facilitator: interaction*

Interaction between researchers and decision-makers was identified as a facilitator of evidence use. Opportunities for direct contact and communication between researchers and decision-makers were found to facilitate evidence-informed decision-making [19,26]. Sustained dialogue [15,26,27] and developing partnerships [28] between researchers and decision-makers were also identified as facilitators of evidence use. Participants in one study suggested, “one-to-one interaction with the researcher to discuss findings, their potential implications for practice, and the opportunity to brainstorm implementation strategies would greatly influence their use of research evidence” [26], p. 159.

## Discussion

This review fills a gap in the literature by synthesizing recent evidence on barriers and facilitators of evidence use at the program management level. To date, the literature has focused largely on decision-making at the clinical [29] and policy levels [30,31]. Despite using a broad search strategy to accommodate for the lack of a standard definition of program management in the literature, the authors were able to identify only 14 empirical studies focused specifically on the use of evidence in decision-making for program management during the time period searched. While reviews addressing program management decision-making exist, often, they also include policy decision-making, precluding examination of barriers and facilitators specifically experienced by individuals involved in the former. For example, a thematic analysis of the recent Orton et al. [31] review of public health decision makers revealed similarities in the types of barriers and facilitators identified. However, a greater emphasis on the ‘Interaction’ theme was found. This could be due to the inclusion of policy-makers, since more weight was given to the influence of researcher-policy-maker interaction as a strategy to promote evidence use at the policy, rather than program management, level [32]. An earlier review of health policy decision-making, which examined evidence from 1966 to 2000, reported barriers and facilitators in the themes of Information, Organization (Structure and Process), and Interaction [30]. A recent review of clinical level decision-making found that clinicians experienced some of the same barriers and facilitators, including the themes of Information, Organization (Structure and Process), Organization (Culture), and Individual Skills [29]. However, a key difference between program managers and both clinicians and policy makers was an emphasis on organizational processes for planning and integrating evidence into decision-making. Therefore, strategies aimed at creating an evidence-informed culture would need to ensure that such processes are appropriately incorporated. To promote evidence use by program managers, such strategies would also need to be directed not only at decision-makers, but also at researchers. Decision-makers value research on complex interventions but experience challenges in the use of evidence to inform decisions due to the absence of a clear definition of evidence. Strategies aimed at improving dissemination and communication of research could increase evidence-use. Both the relevance and timeliness of research could be improved through the exploration of participatory research methods with integrated feedback mechanisms.

Findings from the included studies suggest that decision-makers in health care organizations experience barriers to using evidence at both the organizational and individual level and that efficient ways of integrating evidence-informed decision-making into organizational processes is required. Managers need organizational leaders to not only support them in using evidence, but also to address human resource challenges that inhibit evidence-use. Evidence use could be further increased through the development and implementation of formal organizational processes for decision-making and organizational investment in systems to support evidence-use. In addition, improvements to internal communication mechanisms and processes within organizations and a demonstrated commitment to evidence development and sharing across the organization could be made.

While addressing barriers to evidence-use associated with organizational culture requires executive leadership, those at the individual level require strategies directed at individual skill building. Opportunities for increased interaction between researchers and decision-makers would also serve to promote evidence use.

### Strengths and limitations

A key strength of this review is its focus on program management decision-making. In addition, the inclusion of empirical studies without limiting by study design is another strength. A major limitation of this review is the broad search strategy that was employed. The lack of a standard definition of program management in the literature led the authors to include a broad range of search terms, which ultimately resulted in the identification of a large number of studies not on the topic of interest for this review. Future reviews would benefit from a narrower search strategy. Other limitations of this review include the fact that it was limited to the English language. Therefore, some relevant studies published in other languages may have been excluded. The date limit imposed may also have resulted in relevant studies being excluded. While the reason for limited country representation and Canadian dominance across included studies is not clear, it may be due to an emphasis on evidence-informed decision-making at the program management level by funding agencies in Canada over the last decade. In addition, there may have been differences in the terminology used to describe these activities in other countries. However, the search strategy included a broad range of terms, reducing the likelihood that this was the case.

## Conclusions

The findings from this review of 14 studies exploring the use of evidence in decision-making in program management suggest that barriers and facilitators to evidence use in program management decision-making within health care organizations can be categorized into four distinct groups: (1) Informational, (2) Organizational, (3) Individual, and (4) Interactional. Understanding the barriers and facilitators to evidence-use experienced by managers is an essential first step in developing strategies to promote such evidence-informed decision-making within health care organizations. Health care organizations seeking to improve evidence-informed decision-making by their program managers could use this comprehensive list of barriers and facilitators to identify and address their organization-specific challenges. The findings also confirm that evidence-informed management requires more than encouraging research utilization within organizations. To address informational barriers to evidence-use experienced by managers various sources of evidence need to be considered at different times throughout the decision-making process [33]. Research to determine effective strategies to address organizational barriers to evidence-informed decision-making has yet to be undertaken. Currently, there are gaps in the understanding of the process managers use to apply evidence in health care organizations and how that process can be enhanced to promote evidence-informed decision-making [2,34]. The findings of the review also suggest that strategies to promote evidence use need to be directed individually towards both researchers and decision-makers to enhance the ability of individuals to participate in and promote evidence-informed decision-making. Strategies to foster interaction between researchers and decision-makers should also be explored. The barriers and facilitators of evidence use in decision-making at the management level within health care organizations identified through this review can be used to develop the required multidimensional solutions for promoting evidence-informed program management within health care organizations.

## Competing interests

The authors declare that they have no competing interests.

## Authors’ contributions

SH participated in the design of the study, selection of the articles for inclusion, data abstraction, data analysis and interpretation and drafted the manuscript. TS participated in the design of the study, data abstraction and interpretation and helped draft the manuscript. ZM participated in data interpretation and helped draft the manuscript. DM participated in the design of the study, selection of the articles for inclusion, data analysis and interpretation and helped draft the manuscript. All authors read and approved the final manuscript.

## Authors’ information

Senior Author: Devidas Menon.

## Pre-publication history

The pre-publication history for this paper can be accessed here:

http://www.biomedcentral.com/1472-6963/14/171/prepub

## Supplementary Material

Additional file 1 Literature search strategy. Click here for file

## References

[B1] Hibberd JM Smith DL Nursing Leadership and Management in Canada 2006 3 Canada: Elsevier

[B2] Best A Terpstra JL Moor G Riley B Norman CD Glasgow RE Building knowledge integration systems for evidence-informed decisions J Organ Manag 2009 14 627 641 10.1108/14777260911001644 20020596

[B3] Baker GR Ginsberg L Langley A Champagne F An organizational science perspective on information, knowledge, evidence, and organizational decision-making Using Knowledge and Evidence in Health Care: Multidisciplinary Approaches 2004 Toronto, ON: University of Toronto Press 95 123

[B4] Walshe K Rundall TG Evidence-based management: From theory to practice in health care Milbank Q 2001 14 429 457 10.1111/1468-0009.00214 11565163 PMC2751196

[B5] Clements D What counts? Interpreting evidence-based decision-making for management and policy. Report of the 6th CHSRF Annual Invitational Workshop, Vancouver, British Columbia: March 11 . 2004 2004 Ottawa: Canadian Health Services Research Foundation (CHSRF) Available: http://www.cfhi-fcass.ca/migrated/pdf/event_reports/2004_workshop_report_e.pdf

[B6] Straus SE Tetroe JM Graham ID Knowledge translation is the use of knowledge in health care decision-making J Clin Epidemiol 2011 14 6 10 10.1016/j.jclinepi.2009.08.016 19926445

[B7] Kitson A Straus SE The knowledge-to-action cycle: Identifying the gaps Can Med Assoc J 2010 14 E73 E77 10.1503/cmaj.081231 19948812 PMC2817340

[B8] Higgins JP, Green S Cochrane handbook for systematic reviews of interventions Version 5.1.0 [updated March 2011]. [n.s.] 2011 2011: The Cochrane Collaboration Available: http://www.cochrane.org/handbook

[B9] Canadian Health Services Research Foundation Health services research and…evidence-based decision-making 2000 Ottawa. ON: Canadian Health Services Research Foundation

[B10] Popay J Roberts H Sowden A Petticrew M Arai L Rodgers M Britten N Roen K Duffy S Guidance on the conduct of narrative synthesis in systematic reviews: a product from the ESRC methods programme 2006 Lancaster (UK): Lancaster University Available: http://www.lancaster.ac.uk/shm/research/nssr/research/dissemination/publications/NS_Synthesis_Guidance_v1.pdf

[B11] Pluye P Robert E Cargo M Bartlett G O'Cathain A Griffiths F Boardman F Gagnon MP Rousseau MC Proposal: a mixed methods appraisal tool for systematic mixed studies reviews Mixed methods appraisal tool (MMAT) version 2011 2011 Montreal: McGill University, Department of Family Medicine Available: http://mixedmethodsappraisaltoolpublic.pbworks.com/f/MMAT%202011%20criteria%20and%20tutorial%202011-06-29.pdf

[B12] Dixon-Woods M Bonas S Booth A Jones DR Miller T Sutton AJ Shaw RL Smith JA Young B How can systematic reviews incorporate qualitative research? A critical perspective Qual Res 2006 14 27 44 10.1177/1468794106058867

[B13] Synthesizing Qualitative Research Synthesizing Qualitative Research 2012 Oxford: John Wiley & Sons, Ltd

[B14] Moher D Liberati A Tetzlaff J Altman DG The PRISMA Group Preferred reporting items for systematic reviews and meta-analyses: The PRISMA Statement PLoS Med 2009 14 e1000097 10.1371/journal.pmed.1000097 19621072 PMC2707599

[B15] Dobbins M Rosenbaum P Plews N Law M Fysh A Information transfer: what do decision makers want and need from researchers? Implement Sci 2007 14 20 Available: http://www.ncbi.nlm.nih.gov/pmc/articles/PMC1929120/#__ffn_sectitle 10.1186/1748-5908-2-20 17608940 PMC1929120

[B16] Farmer J Chesson R Health care management: models for evidence-based practice J Manag Med 2001 14 266 282 10.1108/02689230110403777 11765312

[B17] Higgins JW Strange K Scarr J Pennock M Barr V Yew A Drummond J Terpstra J “It’s a feel. that’s what a lot of our evidence would consist of”: public health practitioners’ perspectives on evidence Eval Health Prof 2011 14 278 296 10.1177/0163278710393954 21224264

[B18] Mitton C Patten S Evidence-based priority-setting: what do the decision-makers think? J Health Serv Res Policy 2004 14 146 152 10.1258/1355819041403240 15272972

[B19] Niedzwiedzka BM Barriers to evidence-based decision making among Polish health care managers Health Serv Manage Res 2003 14 106 115 10.1258/095148403321591429 12803950

[B20] Weatherly H Drummond M Smith D Using evidence in the development of local health policies. Some evidence from the United Kingdom Int J Technol Assess Health Care 2002 14 771 781 12602078 10.1017/s0266462302000582

[B21] Wilson MG Rourke SB Lavis JN Bacon J Travers R Community capacity to acquire, assess, adapt, and apply research evidence: a survey of Ontario’s HIV/AIDS sector Impl Sci 2011 14 54 Available: http://www.ncbi.nlm.nih.gov/pmc/articles/PMC3123230/10.1186/1748-5908-6-54 PMC3123230 21619682

[B22] Bowen S Erickson T Martens PJ Crockett S More than “using research”: the real challenges in promoting evidence-informed decision-making Healthc Policy 2009 14 87 102 19377360 PMC2653695

[B23] McDiarmid M Kendall S Binns M Evidence-based administrative decision making and the Ontario hospital CEO: information needs, seeking behaviour, and access to sources JCHLA 2007 14 63 72 10.5596/c07-019

[B24] Dobbins M Cockerill R Barnsley J Ciliska D Factors of the innovation, organization, environment, and individual that predict the influence five systematic reviews had on public health decisions Int J Technol Assess Health Care 2001 14 467 478 11758291

[B25] Ham C Kipping R McLeod H Redesigning work processes in health care: lessons from the National Health Service Milbank Q 2003 14 415 439 10.1111/1468-0009.t01-3-00062 12941002 PMC2690240

[B26] Dobbins M Jack S Thomas H Kothari A Public health decision-makers’ informational needs and preferences for receiving research evidence Worldviews Evid Based Nurs 2007 14 156 163 10.1111/j.1741-6787.2007.00089.x 17850496

[B27] Belkhodja O Amara N Landry R Ouimet M The extent and organizational determinants of research utilization in Canadian health services organizations Sci Commun 2007 14 377 417 10.1177/1075547006298486

[B28] Jbilou J Amara N Landry R Research-based-decision-making in Canadian health organizations: A behavioural approach J Med Syst 2007 14 185 196 10.1007/s10916-007-9054-3 17622021

[B29] Solomons NM Spross JA Evidence-based practice barriers and facilitators from a continuous quality improvement perspective: an integrative review J Nurs Manag 2011 14 109 120 10.1111/j.1365-2834.2010.01144.x 21223411

[B30] Innvaer S Vist G Trommald M Oxman A Health policy-makers’ perceptions of their use of evidence: a systematic review J Health Serv Res Policy 2002 14 239 244 10.1258/135581902320432778 12425783

[B31] Orton L Lloyd-Williams F Taylor-Robinson D O’Flaherty M Capewell S The use of research evidence in public health decision making processes: systematic review PLoS One 2011 14 e21704 10.1371/journal.pone.0021704 21818262 PMC3144216

[B32] Mitton C Adair CE McKenzie E Patten S Perry BW Knowledge transfer and exchange: Review and synthesis of the literature Milbank Q 2007 14 729 768 10.1111/j.1468-0009.2007.00506.x 18070335 PMC2690353

[B33] Scott C Seidel J Bowen S Gall N Integrated health systems and integrated knowledge: Creating space for putting knowledge into action Health Care Q 2009 14 30 36 10.12927/hcq.2009.21094 20057246

[B34] Greenhalgh T Robert G Macfarlane F Bate P Kyriakidou O Diffusion of innovations in service organizations: Systematic review and recommendations Milbank Q 2004 14 239 244 10.1111/j.0887-378X.2004.00325.x PMC2690184 15595944

