# Supplementary material for: Barriers and facilitators to evidence-use in program management: a systematic review of the literature
Source: BMC Health Serv Res. 2014 Apr 14;14:171. doi: 10.1186/1472-6963-14-171 (PMC4101853; doi:10.1186/1472-6963-14-171)
Supplement: Additional file 1 — Literature search strategy. [file 1472-6963-14-171-S1.pdf]

Additional file 1 – Literature Search Strategy

Literature Search Strategy

Dates Conducted: October 2011 – February 2012

Limits: English language only, human studies, 2000 to date

Topic: Strategies within health organizations for capacity building for the use of evidence in program planning. For example, what's effective in promoting evidence use within organizations, use of evidence in context & for program evaluation. Not clinical evidence, policy or program planning evidence. Three concepts within this topic:

- KT within health care organizations
- organizational development
- program evaluation

Concept 1	Concept 2	Concept 3	Concept 4 NOT
MeSH terms	MeSH terms	Title words	
Decision making[majr]	Evidence-based medicine[mh]	Implement*[ti]	Patient[ti]
Decision making, organizational[mh]	Evidence-based practice[mh]	Integrat*[ti]	Physician*[ti]
Policy making [mh]	Knowledge[majr]	Barrier*[ti]	Clinical[ti]
Program development [mh]	Information dissemination[mh]	Uptake[ti]	Nurs*[ti]
Health policy [mh]	Communication barriers[mh]	Facilitat*[ti]	Clinician*[ti]
		Capacity[ti]	Surgical[ti]
Program evaluation[mh]	Interdisciplinary communication[mh]	"evidence use"[ti]	Care[ti]
Health plan implementation[mh]	Persuasive communication[mh]	"research utilisation"[ti]	Community[ti]
Delivery of health Care/methods	Health services research/utilization	"research utilization"[ti]	Therap*[ti]
Models, organizational[mh]	Health services research/organization & administration	"research use"[ti]	Midwife*[ti]
Quality assurance, Health care[mh]	Diffusion of innovation[majr]	"research evidence"[ti]	Child*[ti]
Organizational	Group	"evidence-based"[ti]	Immigrant*[ti]

policy[majr]	processes[mh]		
Systems integration[majr]	Translational research[mh]	“evidence informed”[ti]	Physical[ti]
	Organizational case studies[mh]	Gap[ti]	Family[ti]
Organization and administration/standards	Organizational culture[mh]	Influenc*[ti]	Women*[ti]
		Partnership*[ti]	Adolescent*[ti]
		Improv*[ti]	Medicine[ti]
Additional keywords	Additional keywords		Health promotion[mh]
“program plan*”[ti]	evidence[TI]	“knowledge”[ti]	Emergenc*[ti]
“programme plan*”[ti]	“evidence-based management”[tiab] OR “evidence-informed”[tiab]	“knowledge management”[ti]	Population[ti]
Policy[ti] OR policies[ti] OR policymak*[ti]	“learning organization”[tiab] OR “learning organisation”[tiab]	“knowledge utilization”[ti] OR “knowledge utilisation[ti]	
“decision mak*”[ti] OR decisionmak*[ti]	Manag*[ti]	“knowledge use”[ti]	
		“knowledge translation”[ti]	
	Research Support as Topic[mh]	“knowledge broker*”[ti]	
	Health knowledge, attitudes, practice[mh]	Informed[ti]	
	“research to practice”[ti]	Culture[ti]	
	“best practice*”[ti]	“evidence utilisation”[ti]	
		“utilisation of evidence”[ti]	
		“utilization of evidence”[ti]	

Final search strategy

1. PubMed (www.pubmed.gov, searched 20 Oct 2011) =9783 references

* search term Policy making[mh] added 21 Oct 2011 = 391 additional references

#47	Search #45 OR #46	9783
#46	Search #44 Limits: Humans, English, Publication Date from 2000	9647
#45	Search #44 AND (publisher[sb] OR in process[sb] OR pubmednotmedline[sb]) Limits: Publication Date from 2000	136
#44	Search #35 NOT #43 Limits: Publication Date from 2000	11892
#43	Search #38 OR #40 OR #41 OR #42 Limits: Publication Date from 2000	1506058
#42	Search surgical[ti] OR community[ti] OR therap*[ti] OR midwif*[ti] Limits: Publication Date from 2000	294867
#41	Search immigrant*[ti] OR migrant*[ti] OR physical[ti] OR family[ti] OR women*[ti] OR medicine[ti] OR medical[ti] OR health promotion[mh] OR emergenc*[ti] OR population[ti] Limits: Publication Date from 2000	375649
#40	Search child[ti] OR childhood[ti] OR children[ti] OR adolescent*[ti] OR adolescence[ti] Limits: Publication Date from 2000	212574
#38	Search patient*[ti] OR physician*[ti] OR clinical[ti] OR nurs*[ti] OR clinician*[ti] Limits: Publication Date from 2000	763903
#37	Search #36 Limits: Publication Date from 2000	28767
#36	Search #17 AND (#33 OR #34)	37122
#35	Search #17 AND #34	23895

#34	Search #18 OR #19 OR #20 OR #21 OR #22 OR #23 OR #24 OR #25 OR #26 OR #27 OR #28 OR #29 OR #30 OR	249445

	#31 OR #32	
#32	Search "learning organization"[tiab] OR "learning organisation"[tiab]	139
#31	Search "evidence-informed"[tiab]	305
#30	Search "evidence-based management"[tiab]	516
#29	Search evidence[ti]	146188
#28	Search translational research[mh]	1318
#27	Search diffusion of innovation[mh]	12903
#26	Search health services research/organization & administration	8748
#25	Search health services research/utilization	209
#24	Search persuasive communication[mh]	2501
#23	Search interdisciplinary communication[mh]	6785
#22	Search communication barriers[mh]	3988
#21	Search information dissemination[mh]	7376
#20	Search knowledge[mh] OR knowledge[ti]	35410
#19	Search evidence-based practice[mh]	46870
#18	Search evidence-based medicine[mh]	43280
#17	Search #1 OR #2 OR #3 OR #4 OR #5 OR #6 OR #7 OR #8 OR #9 OR #10 OR #11 OR #12 OR #13 OR #14 OR #15 OR #16	355173

#16	Search "decision making"[ti] OR "decision maker*"[ti] OR decisionmak*[ti]	11913
#15	Search policy[ti] OR policymak*[ti]	25016
#14	Search organization and administration/standards	53010
#13	Search organizational culture[mh]	10759
#12	Search organizational case studies[mh]	7958
#11	Search systems integration[majr]	2205
#10	Search organizational policy[majr]	3030
#9	Search quality assurance, health care[majr]	100604
#8	Search models, organizational[mh]	12729
#7	Search delivery of health care/methods	9179
#6	Search health plan implementation[mh]	2831
#5	Search program evaluation[mh]	46786
#4	Search health policy[mh]	69711
#3	Search program development[mh]	18929
#2	Search decision making, organizational[mh]	9953
#1	Search decision making[majr]	41845

2. The Cochrane Library (John Wiley, issue of 12 2011)

Cochrane Reviews [35] | Other Reviews [3] | Clinical Trials [339] | Methods Studies [191] | Technology Assessments [3] | Economic Evaluations [0] | Cochrane Groups [0]

#1	(decision making):ti,ab,kw or (decision*):ti	4471
#2	(inform OR informed):ti,ab,kw or "evidence based practice":ti,ab,kw or "evidence based management":ti,ab,kw or (research):ti,ab,kw	44386
#3	"learning organization":ti,ab,kw or (information dissemination):ti,ab,kw or (knowledge):ti,ab,kw or (communication):ti,ab,kw or (barriers):ti,ab,kw	17260
#4	(uptake):ti,ab,kw or (diffusion):ti,ab,kw or (integrat*):ti,ab,kw or (implement*):ti,ab,kw or (capacity):ti,ab,kw	32478
#5	(#1 AND #2 AND (#3 OR #4))	571

3. Centre for Reviews and Dissemination (CRD) databases (DARE, HTA, NHS EED)

(http://www.crd.york.ac.uk/crdweb/; searched 3 Nov 2011)

1	MeSH DESCRIPTOR Decision Making, Organizational EXPLODE ALL TREES	15
2	MeSH DESCRIPTOR Decision Making EXPLODE ALL TREES	223
3	"capacity building"	8
4	("learning organization")	1
5	MeSH DESCRIPTOR Information Disse[illegible]ination EXPLODE ALL TREES	19
6	(knowledge):TI OR (communication):TI OR (barriers):TI OR (uptake):TI OR (integrat*):TI	322

7	#1 OR #2	238
8	#5 OR #6	339
9	#7 AND #8	5
10	#3 OR #4 OR #9	14
11	("evidence informed") OR ("research evidence") OR ("knowledge transfer")	215
12	#10 OR #11	228
13	* FROM 2000 TO 2011	38837
14	#12 AND #13	171

4. EMBASE (Ovid, 1980 to 2011 Week 43)

1	exp decision making/	110862
2	policy making.mp. or exp management/	552172
3	learning organization.mp.	128
4	1 or 2	651705
5	exp information dissemination/	9961
6	exp knowledge management/	418
7	barrier*.ti.	29033

8	uptake.ti.	53613
9	evidence informed.mp.	327
10	research evidence.mp.	2888
11	evidence based management.mp.	617
12	knowledge transfer.mp.	648
13	5 or 6 or 7 or 8 or 9 or 10 or 11 or 12	96954
14	4 and 13	3848
15	3 or 14	3974
16	limit 15 to (english language and yr="2000 -Current")	3290
17	(child or childhood or children or adolescent* or adolescence).ti.	598693
18	(immigrant* or migrant* or physical or family or women* or medicine or medical or health promotion or emergenc* or population).ti.	885129
19	(surgical or community or therap* or midwif*).ti.	851196
20	(patient* or physician* or clinical or nurs* or clinician*).ti.	2060046
21	17 or 18 or 19 or 20	4049085
22	16 not 21	2181
23	limit 22 to (human and english language and yr="2000 -Current")	1511

5. ProQuest Dissertations & Theses (searched 4 Nov 2011)

128 documents found for: ("decision making" OR decisionmaker* OR "evidence informed" OR "evidence based manage*" OR "evidence based practice" OR "learning organization" OR "learning organisation") AND ("knowledge trans*" OR "research use" OR "research utilization" OR "research utilisation" OR "research uptake" OR "evidence uptake") AND PDN(>1/1/2000) AND PDN(<12/31/2011)

6. Web of Science (Thomson Reuters; Science Citation Index Expanded (SCI-EXPANDED); Social Sciences Citation Index (SSCI); Arts & Humanities Citation Index (A&HCI); Conference Proceedings Citation Index- Science (CPCI-S); Conference Proceedings Citation Index- Social Science & Humanities (CPCI-SSH); Book Citation Index– Science (BKCI-S); Book Citation Index– Social Sciences & Humanities (BKCI-SSH); searched 4 Nov 2011)

4 521 (#3) AND Language=(English)

Databases=SCI-EXPANDED, SSCI, A&HCI, CPCI-S, CPCI-SSH, BKCI-S, BKCI-SSH Timespan=2000-2011

Lemmatization=On

–

3 551 #2 AND #1

Databases=SCI-EXPANDED, SSCI, A&HCI, CPCI-S, CPCI-SSH, BKCI-S, BKCI-SSH Timespan=All Years

Lemmatization=On

–

2 5,537 Topic=("knowledge trans*") OR Topic=("research use") OR Topic=("research utilization") OR Topic=("research utilisation") OR Topic=("research uptake") OR Topic=("evidence uptake")

Databases=SCI-EXPANDED, SSCI, A&HCI, CPCI-S, CPCI-SSH, BKCI-S, BKCI-SSH Timespan=All Years

Lemmatization=On

–

1 153,278 Topic=("decision making" OR decisionmaker* OR "decision maker*" OR decisionmaking) OR Topic=("evidence informed" OR "evidence based manage*" OR "evidence based practice") OR Topic=("learning organization" OR "learning organisation")

Databases=SCI-EXPANDED, SSCI, A&HCI, CPCI-S, CPCI-SSH, BKCI-S, BKCI-SSH Timespan=All Years

Lemmatization=On

7. CINAHL (Nursing & Allied Health) (EBSCOHost; searched 8 Dec 2011)

S5	S1 and S2 and S3<br><br>Limiters - English Language; Published Date from: 20000101-20111231; Exclude MEDLINE records<br>Search modes - Find all my search terms	513
S4	S1 and S2 and S3	1875
S3	TI implement* OR TI integrat* OR TI barrier* OR TI uptake OR TI facilitat* OR TI utiliz* OR TI research use OR TI using OR TI partner* OR TI gap* OR TI informed	106677
S2	evidence OR knowledge OR information dissemination OR learning organization	210511
S1	decision making OR policy making OR program development OR organizational policy OR (organization and administration)	98552

8. ABI Inform (ProQuest; searched 13 Dec 2011)

33 documents found for: (evidence-based practice) AND (decision making OR organizational behavior) AND PDN(>1/1/2000) AND PDN(<12/31/2011)

9. Library and Information Science Abstracts (LISA; searched 13 Dec 2011)

Search Query #4 TI=(evidence* or knowledge or information) and TI=(decision* or organization* or practice*) and health* 123 Published Works results found in LISA: Library and Information Science Abstracts (limit 2000-2012)

Additional sources (searched 8 Dec 2011 – 28 Feb 2012)

- The Campbell Collaboration Library of Systematic Reviews http://www.campbellcollaboration.org/library.php – scanned list of titles of all reviews = 0 relevant references

- KU-UC database http://kuuc.chair.ulaval.ca/english/index.php - scanned titles in category for Evidence-based decision making OR knowledge utilization = 469 references;   7 relevant non-duplicate references added to database

- KT+ database http://plus.mcmaster.ca/kt/Default.aspx - searched keywords “decision*” and “evidence”; scanned results, references too clinical; nothing relevant identified:

Search Results	
Quality-Filtered Articles*:	View 50 matches
Original Studies Included in Quality Filtered Reviews†:	View 152 matches
Quality Improvement Studies from Pubmed‡:	View 100 matches

- NYAM Grey literature collection http://www.nyam.org/library/online-resources/grey-literature-report/ - searched “decision making” = 150 hits, 4 possibly relevant; “decision” AND “evidence” = 59 hits, 1 possibly relevant
- MEDLINE Author Search
  16 “Lavis JN” [Author] 98
  15 lavis j 124

14 “Lomas J” [Author]	124
13 Lomas J	167
12 “Oxman AD” [Author]	193
11 oxman ad	193
10 “Kovner AR” [Author	42
9 Kovner ar	42
8 “Rundall TG” [Author]	56
7 rundall tg	56
6 “Grimshaw JM” [Author]	132
5 grimshaw jm	378
4 “Dobbins M” [Author]	40
3 dobbins m	75
2 “Ham C” [Author]	181
1 ham c	202
